# Health seeking behaviour and delayed management of tuberculosis patients in rural Bangladesh

**DOI:** 10.1186/s12879-018-3430-0

**Published:** 2018-10-12

**Authors:** K. A. T. M. Ehsanul Huq, Michiko Moriyama, Khalequ Zaman, Mohammod Jobayer Chisti, Julie Long, Akramul Islam, Shahed Hossain, Habiba Shirin, Mohammad Jyoti Raihan, Sajeda Chowdhury, Md Moshiur Rahman

**Affiliations:** 1 Graduate School of Biomedical & Health Sciences, Hiroshima University, Kasumi 1-2-3 Minami-ku, Hiroshima, 734-8553 Japan; 2 International Centre for Diarrhoeal Disease Research, Dhaka, Bangladesh; 3 University of Colorado, Denver, USA; 4 Health Programme, BRAC, Dhaka, Bangladesh; 5 Institute of Biomedical & Health Sciences, Hiroshima University, Hiroshima, Japan

**Keywords:** Tuberculosis, Health seeking, Delay, Health care provider, Bangladesh

## Abstract

**Background:**

Early diagnosis of tuberculosis (TB) and involvement of the public-private partnership are critical to eradicate TB. Patients need to receive proper treatment through the National Tuberculosis Control Programme (NTP). This study describes various predictors for health seeking behaviour of TB patients and health system delay made by the different health care providers.

**Methods:**

A cross-sectional study was conducted in a public health facility of a rural area in Bangladesh. Newly diagnosed smear positive pulmonary TB (PTB) patients who were ≥ 15 years of age were sequentially enrolled in this study. The socio-demographic characteristics and proportion of health care utilization by the patients, and health system delay made by the health care providers were calculated. Multivariate analysis was conducted to determine the independent association of the risk factors with the time to seek medical care.

**Results:**

Two hundred and eighty patients were enrolled in this study. Among them, 73.6% were male and 26.4% were female. A hundred percent of patients primarily sought treatment for their cough, 170 (60.7%) first consulted a non-qualified practitioner while 110 patients (39.3%) first consulted with qualified practitioners about their symptoms. Pharmacy contact was the highest (27.9%) among the non-qualified practitioners, and 58.9% non-qualified practitioners prescribed treatment without any laboratory investigation. The average health system delay was 68.5 days. Multiple logistic regressions revealed a significant difference between uneducated and educated patients (OR 2.33; CI 1.39–3.92), and qualified and non-qualified practitioners (OR 2.34; CI 1.38–3.96) to be independent predictors of health system delay.

**Conclusions:**

Compared to men, fewer women sought TB treatment. Uneducated patients and questionably qualified practitioners made for a longer delay in detecting TB. Increasing public health awareness and improving health seeking behavior of females and uneducated patients, and greater participation of the qualified practitioners in the NTP are highly recommended.

**Electronic supplementary material:**

The online version of this article (10.1186/s12879-018-3430-0) contains supplementary material, which is available to authorized users.

## Background

Tuberculosis (TB) is a major public health problem and is, globally, the ninth leading cause of death. In 2016, of the 10.4 million new cases worldwide, 1.3 million resulted in death [1]. This infectious disease is most commonly spread by coughing [2] and is most prevalent among productive-age adults between 15 and 59 years [3]. According to ‘The End TB Strategy’ in the Sustainable Development Goals (SDGs), early diagnosis of TB and engagement of public and private health care providers is essential [1, 4]. To reduce the case fatality rate from 16 to 10% between 2015 and 2020, it is necessary to diagnose and treat patients without any delay [1]. Patients who are not diagnosed and treated early, act as reservoirs for spreading the disease [5]. Access to TB services for diagnosis, treatment and prevention is necessary to promote and protect human rights and gender equalities [6]. Currently, the world is experiencing a pulmonary TB epidemic. The increased incidence of multi-drug resistant tuberculosis has contributed to the increased global burden of TB [7].

The chance of catching the infection depends on the close contact, frequency and duration of exposure with TB patients [8]. Delayed diagnosis and close contact with a TB patient significantly increase the risk of transmission of infection [9]. Moreover, delays in TB diagnosis may cause a significant adverse outcome, which may increase fatality rates [10]. A systematic review found that the average health system delay for treating TB was nearly the same in developing and developed countries (28.5 and 21.5 days respectively) [11]. The public health response to the increased threat of TB in the community is to prevent the transmission of TB [10, 12]. This is achievable by treating active TB patients and screening and using chemoprophylaxis for those who come in contact with active cases [10]. However, a systematic review found that in many countries, more than half of the TB cases remain undiagnosed in the community despite active surveillance [12].

Universal access to quality treatment for all TB patients is necessary to achieve the goal of a TB free world [1]. Most countries now provide free treatment facilities through the National Tuberculosis Control Programme (NTP). NTP is normally implemented by the government health services of a country’s national health system. However, many private health care providers and non-government organizations (NGOs) are also involved in the NTP delivery system [13, 14].

In many settings, patients with symptoms like persistent cough, have an equal opportunity to utilize both public health care providers within the NTP as well as private providers, such as drug sellers and traditional healers, who are not under the NTP [13]. Because of the weak health infrastructure in some countries, patients seek care outside the NTP. Moreover, some public central hospitals that provide health services for prisoners, police and army personnel are also not covered by the NTP [13]. While still, some countries based their official reports only on information about patients within NTP facilities, resulting in a risk of under-reporting patients who attend facilities outside the NTP. Despite the availability of standard NTP treatment, patients may utilize private providers and receive inadequate treatment, which contributes to the development of multi-drug resistance TB [13]. In many developing countries, like Bangladesh, most TB patients’ first contact with a private physician is at the primary health care facility of their choice, which is not directly linked with the NTP [15]. Pharmacists and drug sellers who specifically treat poor patients may not be aware of the standard guidelines for proper TB management and the referral system. They are not the part of NTP and have no specific training on TB management [15].

With the public facilities, TB patients also practiced self-medication, and went to private practitioners and traditional healers for their primary consultation. Patient’s lack of awareness about TB symptoms and lack of free treatment facilities in the public health care facilities and dissatisfaction with the public health care systems resulted in more delays [16]. It is noteworthy that public-private partnership could improve TB case notifications, treatment success rates, decreased defaulter patients, service accessibility and increase referral system of TB patients to the NTP [17]. In the End TB Strategy of WHO, all care providers are included in the NTP [1]. In underprivileged countries, the TB control programme is often more effective than other health services due to its highly-trained staff, uninterrupted drug supply, proper diagnostic and treatment facilities, a good reporting system and, most importantly, all services are free of charge [13]. Most of the countries with a high TB burden allocate a large portion of the national health budget to the TB control program. Additional funds are provided from the Global Fund according to the single stream funding policy for the sustainability of the TB project [1, 18]. Despite an adequately financed and effective TB management programme that exist in Bangladesh, the morbidity and mortality from TB are still high [1]. The incidence rate was almost static between 2000 and 2016; moreover, the notifications of new and relapse cases increased significantly over that period of time [1]. Compared to massively growing urban areas, NTP is more effective in the rural areas. Overcrowded and high density residences, inadequate and packed public transportation make for easy transmission of TB. Poor people, particularly those moving from rural areas to urban areas, live in extremely overcrowded slums and they move from one slum to another with shorter intervals, which impacts adversely on their access to health facilities and proper treatment. Consequently, the prevalence of TB is significantly higher in urban areas [19].

Understanding of TB patients’ health care seeking patterns is helpful for improving the organization of the National Tuberculosis Program (NTP), which in turn improves early detection of TB and helps patients receive proper treatment without delay. The primary aim of this study was to explore the patterns of health care utilization by TB patients before starting treatment at a government health care facility in rural Bangladesh. As a secondary objective, the study attempted to shed light on the relationship between different factors such as demographic and socio-economic characteristics and treatment delays within the health system.

## Methods

### Study design and study population

This was a cross-sectional study. The study was conducted in a rural area called Matlab Upazila in Bangladesh about 55 km from the capital, Dhaka. The population of this community was 227,853, with 46% being male and 54% female and 68% of the population was aged ≥15 years in 2013. Thirty percent had no formal education, and 86% were Muslim. Male and female literacy was 75 and 67%, respectively. The primary sources of income were from agriculture, other daily labour, and small business. The coverage of the Bacillus Calmette-Guerin (BCG) vaccine for the prevention of tuberculosis was 97.9% or more [20, 21]. All the TB patients were prospectively enrolled from the Upazila (Sub-district) Health Complex (UHC) between April 2005 and May 2009 to minimize biases. Patients, who had smear positive pulmonary tuberculosis, were interviewed after diagnosis. We evaluated the health care seeking patterns of the TB patients before commencing treatment at the Upazila Health Complex, in order to assess the delay for the diagnosis of TB.

### Data collection

After the diagnosis of TB (smear positive TB case) at the UHC, patients were invited to participate in the study. A Field Research Assistant (FRA) usually contacted the patient at home on the day following the diagnosis, and a voluntary informed consent form (ICF) was signed. The FRA also administered a structured questionnaire to collect required study information. Both the ICF and the questionnaire were translated into Bengali from English. Study procedures took 30–40 min to complete per participant. The questionnaire contained questions regarding the type of health seeking and utilization patterns, referral attitude and action of the health care providers, use of health care providers, reasons for changing provider, time taken to receive treatment at the UHC, knowledge about the free treatment facilities, and socio-demographic characteristics of the patients. The time taken to receive treatment (health system delay) was calculated using data about type and number of health care providers the patient visited before the diagnosis of TB at UHC.

### Diagnosis and treatment

In Bangladesh, each Upazila Health Complex has around 30 to 50 beds, including both indoor and outdoor facilities. In the Matlab Upazila Health Complex (UHC) all suspected TB cases with a history of cough for more than 3 weeks were seen by a study physician. The physician performed a physical examination and gave advice regarding laboratory testing. UHC has facilities to examine sputum for acid-fast bacilli (AFB), perform chest X-rays and other routine examinations. Three sputum spot samples (overnight, morning, and following day) were collected (about 5 ml per sample) for microscopic examination for the AFB test. Sputum samples were collected and examined by the UHC laboratory technician. Standard guidelines were followed for the diagnosis and treatment in accordance with the National Tuberculosis Control Programme algorithm [22].

### Sample size calculation

We assumed the proportion of smear positive TB patients seeking care from qualified and non-qualified health care providers was 25 and 75%, respectively [23]. The calculated sample size was 288 with a 95% confidence interval and 5% error [24]. It took 4 years to complete the study. All sputum smear positive patients who attended the Matlab Upazila Health Complex were enrolled. According to UHC records, approximately 75 TB patients were diagnosed each year for a total of nearly 300 smear positive cases during the study period.

### Operational definitions

A systematic review, analyzing 52 studies, found that the delay in treatment could be stratified by patients’ delay, health system delay and total delay [11]. Health system delay was considered as a delay between the first consultation with a formal or informal provider and the diagnosis of TB. This delay ranged from 2 to 87 days. The length of health system delays differed depending on whether patients sought treatment from formal or informal health care providers [11]. For the purpose of our study, we defined health system delay as the time between first consultation with any health care professional (both qualified and non-qualified practitioner) and the initiation of proper TB treatment. Based on the previous study in Bangladesh, we calculated the mean health system delay to be ≥45.6 days [25]. Considering the health system delay, we have recognized “considerable delay” to be 45.6 days and above and “acceptable delay” to be less than 45.6 days for our study. Moreover, in our analyses, proper ‘action taken’ is defined as referral to UHC. Whereas, ‘qualified practitioner’ is defined as a medical doctor or a special government-training certificate for TB management who worked at the UHC.

### Data analysis

All questionnaires were checked for validity, consistency, and completeness. After tabulating the data, frequency distributions were checked for each variable to identify any abnormalities or extreme values. With respect to ‘delay’ in using health care system by the TB patients, we estimated the odds ratios with 95% CIs. All key variables used are mentioned in Tables 1 and 2. Finally, using the significant predictors of considerable delay, multivariate analyses in the form of a logistic regression were performed to reflect on the relationship between individuals’ background characteristics, and considerable delay with adjustment for other variables. Data analyses were performed using software package SPSS version 17.0 (SPSS Inc., Chicago, USA). One US dollar (USD) was considered equivalent to 76 Bangladeshi Taka (BDT).

**Table 1:** Distribution of age, family size and monthly income

Items	Male	Female
	*n* (%)	*n* (%)
Age:		
15–45 years	69 (33.5)	46 (62.2)
> 45 years	137 (66.5)	28 (37.8)
Total	206 (73.6)	74 (26.4)
Family size and monthly income		
	Mean (SD)	Median (IQR)
Family size mean (SD)	5.37 (2.3)	5.0 (4.0–6.7)
Average monthly income (BDT)	4312.5 (2281.6)	4000 (3000–5000)

*SD* Standard deviation, *IQR* Interquartile range, *BDT* Bangladesh Taka

**Table 2:** Basic demographic characteristics of this study

Items	*n*	%
Religion		
Muslim	241	86.1
Hindu	39	13.9
Occupation		
Agriculture	59	21.1
Day Laborer	22	7.9
Fisherman	8	2.9
Boatman	2	0.7
Service	12	4.3
Teacher	2	0.7
Rickshaw puller	11	3.9
Business	41	14.6
Student	5	1.8
Doctor/Engineer/Lawyer	1	0.4
Unemployed	93	33.2
Driver	1	0.4
Garment/Factory worker	2	0.7
Others	21	7.5
Received BCG vaccine		
No	233	83.2
Yes	47	16.8
Smoking habits		
No	87	31.1
Yes	193	68.9
Duration of Smoking		
≤ 10 Years	55	28.5
> 10 Years	138	71.5
History of TB in Family		
Yes	117	41.8%
No	163	58.2%

*BCG* Bacillus Calmette-Guerin, *TB* Tuberculosis

## Results

In accordance with the sample size estimation, 280 patients were diagnosed with pulmonary TB-they all consented and were successfully interviewed. Two hundred and six patients (73.6%) were male and 74 (26.4%) were female. Most (66.5%) of the male patients were older than 45 years of age, but most (62.2%) of the female patients were between 15 and 45 years of age. The basic demographic characteristics of the patients suggest that their average household size was 5.4 ± 2.3 with a median (range) of 5.0 (4.0–6.7). The average monthly household income was 4312.5 BDT and 198 out of the 280 (70.7%) patients lived below the poverty line. The maximum monthly income was BDT 20,000, and minimum was BDT 1000, reflecting a disparity in the community (Table 1).

Among the patients, 86.1% were Muslims and 13.9% were Hindus; 43.6% had no formal education, 33.2% had 1-5 years, 20.4% had 6-10 years, and only 2.9% had more than 10 years of formal education. In terms of occupation, 33.2% were unemployed; and among the employed most (21.1%) were involved in the agriculture. The data indicated that 83.2% had not received the BCG vaccine, 68.9% were smokers (with 71.5% of smokers smoking for more than 10 years) and 41.8% of the patients had a history of TB in the family (Table 2).

**Fig. 1:**
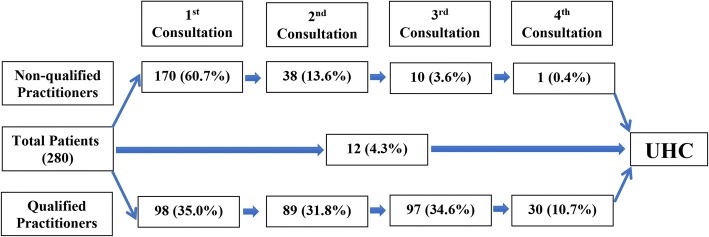
Flow chart of patients’ consultation for non-qualified and qualified practitioners. *Note*: the dropout patients directly consulted at Upazila Health Complex (UHC)

All (100%) patients were seeking treatment for their cough. Among the patients, 170 (60.7%) patients had first consulted a non-qualified practitioner while 110 (39.3%) had consulted a qualified practitioner about their symptoms. The patients received a different quality of care during consultations from the non-qualified and qualified practitioners before attending the UHC (Fig. 1). Among all those who consulted non-qualified practitioners (170), pharmacy contact was the most common, (78 or 45.9% of all patients who sought care from non-qualified practitioners). The remaining patients had sought treatment from other diverse types of non-qualified practitioners such as licentiate of medical faculty (LMF), village doctors, medical assistants, sub-assistant community medical officers (SACMOs) who are designated helping hands of qualified physicians, and traditional and faith healers. Moreover, of the patients who took advice from non-qualified practitioners, only 31 (18.2%) were referred to the UHC (i.e. proper action taken) (Table 3).

**Table 3:** Distribution of health care providers at first consultation

Items	*n*	(%)
First Consultation		
Non-qualified Practitioner	170	60.7
Pharmacy	78	27.9
Others	72	25.7
NGO Paramedics	18	6.4
Homeopath	1	0.4
Ayurvedic	1	0.4
Qualified Practitioner	110	39.3
Private Practitioner	64	22.9
icddr,b	33	11.8
UHC	12	4.3
DOTS Centre	1	0.4

*NGO* Non-governmental organization, *UHC* Upazila Health Complex, *icddr,b* International Centre for Diarrhoeal Disease Research, Bangladesh, *DOTS* Directly-observed treatment short-course

Among all patients, 165 (58.9%) were prescribed with drugs at the first consultation without performing any laboratory investigation. Seventy-five (26.8%) patients were advised to undergo laboratory investigations, 5 (1.8%) were referred to a TB clinic elsewhere and 2 (0.7%) received advice on cough management only and 1 (0.4%) patient was referred to doctors who were a chest or pulmonary specialist (not shown in table).

Most of the patients (90%) had knowledge/awareness of the free treatment facilities provided by the government and NGOs (BRAC, a private for profit). Moreover, 76.1% patients knew the signs and symptoms of TB. Patients who sought treatment from other health care providers had an average delay of 68.5 days till proper initiation of TB treatment at the UHC (Table 4). Health system delay according to the patients’ demographic characteristics in respect to non-qualified and qualified practitioners is shown in Table 4.

**Table 4:** Health system delay in the initiation of TB treatment among patients first receiving advice from non-qualified or from qualified practitioners

Characteristics	Non-qualified Practitioner	SD	Qualified Practitioner	SD	Overall delay	SD
	Days		Days		Days	
	Mean		Mean		Mean	
All	66.52	80.24	70.90	207.66	68.50	151.61
Age						
≤ 44	67.66	85.58	70.84	97.09	69.07	90.47
> 44	65.70	76.65	70.93	257.22	68.11	182.80
Sex						
Male	61.01	78.13	72.95	234.65	66.57	169.70
Female	80.60	84.70	64.55	81.84	73.88	83.33
Religion						
Muslim	69.36	83.79	78.43	225.94	73.35	162.10
Hindu	45.22	40.93	32.86	42.84	38.56	41.89
Education						
Uneducated	78.72	85.34	74.05	81.92	76.38	84.13
Educated	61.25	57.23	71.81	64.51	66.53	62.87
Income						
≤ 3500	70.07	91.03	71.56	273.58	70.75	173.91
> 3500	58.26	46.13	69.22	100.46	63.07	74.65
BCG vaccination						
No	76.46	89.71	78.81	80.38	77.63	87.10
Yes	64.79	68.34	71.23	74.51	68.01	78.42

*SD* Standard deviation, *BCG* Bacillus Calmette-Guerin

The results of the bivariate analyses suggest that education, BCG vaccination and types of health care providers during the first consultation were significantly associated with the duration of the delay. The results suggest that an acceptable delay was 2.17 times more likely if the patient was educated in comparison to being uneducated. Participants who received the BCG vaccination were 2.13 times likely to have an acceptable delay in comparison with those who were not vaccinated. However, our result shows that patients who consulted qualified personnel were 2.08 times less likely to make an acceptable delay (Table 5).

**Table 5:** Relationship of demographic and socio-economic factors with time delayed

Variable		Accepted delay (< 45.6 days)	Considerable Delay (≥ 45.6 days)	OR (95% CI)
Age	≤ 44	66 (62.3%)	40 (37.7%)	Ref.
	> 44	124 (71.3%)	50 (28.7%)	0.66 (0.34–1.11)
Sex	Male	145 (70.4%)	61 (29.6%)	Ref.
	Female	45 (60.8%)	29 (39.2%)	1.53 (0.88–2.66)
Religion	Muslim	159 (66.0%)	82 (34.0%)	Ref.
	Hindu	31 (79.5%)	8 (20.5%)	0.51 (0.22–1.14)
Patient’s Occupation	Unemployed	21 (80.8%)	5 (19.2%)	Ref.
	Employed	169 (66.5%)	85 (33.5%)	2.11 (0.77–5.80)
Patient’s Education Status	Uneducated	94 (77.0%)	28 (23.0%)	Ref.
	Educated	96 (60.8%)	62 (39.2%)	2.17 (1.27–3.68)
Average monthly income	≤ 3500	82 (69.5%)	36 (30.5%)	Ref.
	> 3500	108 (66.7%)	54 (33.3%)	1.14 (0.68–1.90)
Receiving BCG vaccine	No	165 (70.8%)	68 (29.2%)	Ref.
	Yes	25 (53.2%)	22 (46.8%)	2.13 (1.13–4.04)
Smoking	No	60 (69.0%)	27 (31.0%)	Ref.
	Yes	130 (67.4%)	63 (32.6%)	1.08 (0.62–1.86)
Smoking duration	≤ 15 years	115 (69.7%)	50 (30.3%)	Ref.
	> 15 years	40 (34.8%)	35 (21.2%)	1.23 (0.74–2.04)
Family member who have TB	No	144 (66.7%)	72 (33.3%)	Ref.
	Yes	46 (71.9%)	18 (28.1%)	0.78 (0.42–1.45)
Family member who had TB	No	106 (65.0%)	57 (35.0%)	Ref.
	Yes	84 (71.8%)	33 (28.2%)	0.73 (0.43–1.22)
First Consultation	Non- qualified person	90 (52.9%)	80 (47.1%)	Ref.
	Qualified person	77 (70.0%)	33 (30.0%)	0.48 (0.30–0.80)
Having knowledge and awareness about TB and free TB treatment	No	19 (67.9%)	9 (32.1%)	Ref.
	Yes	171 (67.9%)	81 (32.1%)	1.00 (0.43–2.30)
Who referred at MUHC	Self/Relative	29 (59.2%)	20 (40.8%)	Ref.
	Other	161 (69.7%)	70 (30.3%)	0.63 (0.33–1.19)

*OR* Odds ratio, *CI* Confidence interval, *Ref.* Reference, *BCG* Bacillus Calmette-Guerin, *TB* Tuberculosis, *MUHC* Matlab Upazila Health Complex

Finally, the binary logistic regression model, using the significant predictors of acceptable delay as a dependent variable, patients’ lack of education and first consultation with a qualified service provider were independently associated with the delay in the initiation of proper TB treatment. The result suggests that primarily the odds of an acceptable delay was 2.33 times higher for educated patients and 2.34 times greater if the patient sought care from a non-qualified person (Table 6). Age, sex and income did not confound the size of the effect of the statistically significant variables, as they remained similar when adjusted for age, sex and income level.

**Table 6:** Multivariable analysis showed the associations between independent (x) background factors and dependent (y) variables as acceptable delay

Variable	Categories	OR (95% CI)
Education	Uneducated	Ref.
	Educated	2.33 (1.39–3.92)
BCG Vaccine	Not received	Ref.
	Received	1.41 (0.73–2.71)
First Consultation	Qualified person	Ref.
	Non-qualified person	2.34 (1.38–3.96)

*OR* Odds ratio, *CI* Confidence interval, *Ref,* Reference, *BCG* Bacillus Calmette-Guerin

## Discussion

These findings indicate that patients’ education, BCG vaccination status and first consultation with qualified persons were significant predictors for delay initiation of proper TB treatment. Educated patients who primarily sought care from health care personnel were more likely to have an acceptable delay in starting proper TB treatment in comparison to uneducated patients. Moreover, qualified personnel had longer delays before initiation of TB management. A lower proportion of women were diagnosed with TB at the facility, and they had a higher rate of considerable delay before initiating treatment. For this study, being educated reduces the odds of considerable delay in getting proper treatment of TB. In fact, despite proper diagnosis or guidance during the first consultation, uneducated patients may suffer a considerable delay in the initiation of proper TB treatment. Reasons include time wasted to seek a second or even third opinion due to psychological factors of denial among the patients diagnosed with this stigmatized disease [26–28].

Whereas, in terms of a considerable delay made before initiation of proper TB treatment after the first consultation with qualified personnel, it could be explained that some of the private practitioners with less awareness about TB may not want to acknowledge failure in diagnosing; therefore, they do not refer patients to UHC. This may justify the longer delay made in this sample who first sought care from private practitioners, and similar findings are evident also in another study in Bangladesh [29] and elsewhere [12]. This is often observed, as private practitioners repeatedly prescribe different diagnosis procedures and treatments for cough before referring the patients to UHC, or did not refer patients at all. In this case, the patients sought treatment from other places causing a further delay in the initiation of proper TB treatment (Fig. 1).

The tendency for patients to seek care from private practitioners in this rural setting, was much lower than that observed in another study that covered populations from all over Bangladesh in which 43.8% first sought care from the private practitioners before reporting to public health facilities [29]. Two other studies in Indonesia and India have shown that 27.3 and 48.7% respectively visited private facilities [30, 31].

A study in rural Tanzania [32] observed a somewhat lesser health system delay in the initiation of treatment (57 days) than our study, while the average delay was 151 days in Afghanistan [33] and 16 days in China [34].

Furthermore, the knowledge and awareness of TB in this study population was higher (76.1%) than in the neighboring country of India, where 56% of the people knew about TB and only 20% knew about the cause and mode of TB transmission [35]. Most of our study population did not receive BCG vaccine during their childhood, which indicates that vaccination coverage in this area has increased [20, 21].

Our study found that less women sought treatment than men, though the gender distribution in the catchment was the same, which is in concordance with the national prevalence of TB in Bangladesh (male and female ratio more than 3:1, [1] 1.45:0.48 [25] and findings from other middle and low-income countries [36]. The reasoning for less female attendance may be attributed to the social stigma associated with TB [25, 28]. Women prefer to visit traditional healers and seek alternative medicine rather than mainstream medical health professionals [28]. Even though fewer women consult with qualified health care providers, clinicians are not able to aggressively investigate for the diagnosis of TB [20, 37, 38]. As a result, further evaluation of the low rates of female hospital attendance and TB among women is needed.

It is likely that access to health care was limited due to poor socio-economic conditions of this study population. This might have made the patients choose non-qualified practitioners, especially pharmacists, due to easy access and low-price of drugs. This finding is similar to a WHO report on the National Tuberculosis Control Programme of Bangladesh [15].

This paper suggests that all medical service providers, both qualified and non-qualified, should be integrated into the National Tuberculosis Control Programme (NTP). This integration could improve the proper referral system, increase awareness and reduce the stigma surrounding TB. It may also help to increase the utilization of free and complete treatment facilities for TB provided by the government.

In order to achieve the Sustainable Development Goals (SDGs), TB needs to be further highlighted in the National Health Policy of Bangladesh and incorporated into various public health programmes to increase knowledge, awareness, reduce gender differences and stigma in the National Tuberculosis Programme (NTP).

### Limitations

The limitations of this study were that we excluded all the patients who were less than 15 years, as well as extra-pulmonary TB patients, because it was very difficult to diagnose this age group due to nonspecific clinical symptoms. We also did not enroll those patients who did not visit the Upazila Health Complex, but sought treatment from private health care providers. The study was carried out in a single health facility; therefore, the findings are not representative of the whole of rural Bangladesh. The study delay data relied on patients recall and was thus prone to recall bias. This is a cross-sectional study, and thus no causal inferences could be made. Because of the above-mentioned limitations, the results may not be extrapolated to depict the TB situation in Bangladesh.

## Conclusions

In conclusion, though most patients knew about the signs and symptoms of TB and the existence of free TB treatment facilities, the majority of the patients primarily consulted a non-qualified practitioner for their symptoms. Educated patients and non-qualified practitioners experienced less delay for proper TB treatment. Also, females were less likely to seek treatment. This study has identified a critical knowledge gap regarding patients and their health seeking behavior. This study’s findings suggest that less-educated and female patients need to increase their awareness and more qualified practitioners need to be integrated into the NTP. These discoveries may help assist further the implementation of future health intervention for proper diagnosis and treatment of TB patients.

## Additional file

Additional file 1: Provided the data set that was used for this manuscript. Total 280 patients were enrolled and analyzed and variables used for this manuscript. (XLS 490 kb)

## Abbreviations

AFB: Acid-fast bacilli
BCG: Bacillus Calmette-Guerin
BDT: Bangladesh Taka
Cl: Confidence interval
DOTS: Directly-observed treatment short-course
FRA: Field research assistant
icddr,b: International Centre for Diarrhoeal Disease Research, Bangladesh
ICF: Informed consent form
IQR: Interquartile range
LMF: Licentiate of medical faculty
MUHC: Matlab Upazila Health Complex
NGOs: Non-government organizations
NTP: National Tuberculosis Control Programme
OR: Odds ratio
PTB: Pulmonary tuberculosis
SACMO: Sub-assistant community medical officer
SD: Standard deviation
SDGs: Sustainable Development Goals
SPSS: Statistical package for social sciences
TB: Tuberculosis
UHC: Upazila Health Complex
USD: United State Dollar
WHO: World Health Organization
